# Long-term evaluation of viability of microfilariae and intravenously transplanted adult *Dirofilaria immitis* in microfilaremic dogs treated with low-dose, short- and long-treatment regimens of doxycycline and ivermectin

**DOI:** 10.1186/s13071-023-05769-2

**Published:** 2023-06-09

**Authors:** John Wilson McCall, Abdelmoneim Mansour, Utami DiCosty, Crystal Fricks, Scott McCall, Michael Timothy Dzimianski, Ben Carson

**Affiliations:** 1 TRS Labs, Inc., Athens, GA 30607 USA; 2 Department of Infectious Diseases, College of Veterinary Medicine, University of Georgia, Athens, GA 30602 USA

**Keywords:** *Dirofilaria immitis*, Mosquitoes, Migration, Development, Dogs, Doxycycline, Ivermectin, Blocking transmission

## Abstract

**Background:**

Microfilarial (mf) counts were monitored over 21.3 months for any rebound that might occur in counts, and adulticidal efficacy was assessed following administration of low dosage with short- and long-treatment regimens of doxycycline and ivermectin to heartworm-microfilaremic dogs.

**Methods:**

Twelve heartworm-naïve beagles infected with 10 pairs of adult *Dirofilaria immitis* by intravenous transplantation were randomly allocated to three groups of four dogs. All treatments started on day 0. On day 0, Group 1 (short-treatment regimen) received doxycycline orally at 10 mg/kg once daily for 30 days plus ivermectin orally (minimum, 6 mcg/kg) on days 0 and 30. Group 2 (long-treatment regimen) received doxycycline orally at 10 mg/kg once daily until individual dogs became mf-negative (72–98 days) and ivermectin every other week until individual dogs became mf-negative (6–7 doses). Group 3 was the untreated control. Mf counts and antigen (Ag) tests were conducted. Dogs were necropsied for recovery and enumeration of heartworms on day 647.

**Results:**

Day −1 mean mf counts were 15,613, 23,950, and 15,513 mf/ml for groups 1, 2, and 3, respectively. Mean counts for Groups 1 and 2 declined until days 239 and 97, respectively, when all were negative. Group 3 had high mf counts throughout the study. There was not a rebound in mf counts in any of the treated dogs after they became amicrofilaremic. All dogs in group 1 and group 3 were Ag-positive throughout the study and had at least one live female worm at necropsy. All dogs in treated Group 2 were positive for Ag through day 154, but were antigen-negative on days 644 and 647, as all had only male worms. Mean live adult worm recoveries for Groups 1, 2, and 3 were 6.8 (range, 5–8), 3.3 (range, 1–6), and 16.0 (range, 14–17), respectively, with a percent reduction in adult worm counts of 57.5% for Group 1 and 79.3% for Group 2.

**Conclusions:**

These data lend support to the use of the American Heartworm Society Canine Guidelines for adulticide therapy recommending the initiation of doxycycline plus a macrocyclic lactone (ML) at the time of the heartworm-positive diagnosis.

**Graphical Abstract:**

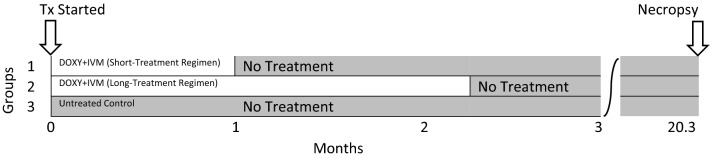

## Background

The endosymbiont *Wolbachia pipientis* (Rickettsiaceae) is present in some of the most important species of filarial parasites, including *Dirofilaria immitis*, *Wuchereria bancrofti*, *Onchocerca volvulus*, *Brugia malayi*, *Brugia pahangi*, *Litomosoides sigmodontis*, and others [1–8]. Although the numbers of *Wolbachia* vary among individuals and life-cycle stages, every individual worm in these filarial species has at least some of the bacteria [7, 9]. *Wolbachia* are needed for normal growth and development of these filariae, and sufficient indirect and direct evidence indicates that the association between the filaria and *Wolbachia* is obligatory, with a strong mutualistic interaction [7]. Earlier work demonstrated that tetracycline drugs had sublethal effects such as inhibition of embryogenesis and infertility against *B. pahangi*, *D. immitis*, *L. sigmodontis*, *O. volvulus* and *W. bancrofti* [2, 3, 10–15], inhibition of third-stage larval development and in vivo prophylaxis against *B. pahangi* and *L. sigmodontis* [10, 11, 16], stunting of adult worm growth in *L. sigmodontis* [11] and *B. pahangi* (McCall et al., unpublished data), and even death of adult worms of *Onchocerca ochengi* in cattle [17], *O. volvulus* in humans [13], and *W. bancrofti* in humans [18]. There is general agreement that the antifilarial effects of tetracycline therapy are a result of activity against *Wolbachia* because these antibiotics have no effect on the *Wolbachia*-negative filariae such as *Acanthocheilonema viteae* [11, 16] and because the antibacterial effect precedes the antifilarial effects [7, 13, 14, 17].

In regard to *D. immitis*, the tetracycline drug doxycycline, administered with or without a macrocyclic lactone (ML) preventive, has been shown to adversely affect every life-cycle stage: circulating microfilariae (mf) [3, 19–26], infective third-stage larvae (L3) and fourth-stage larvae (L4) [23, 27], juveniles [20, 27], young adults [20], and mature adults [19, 21–23, 25, 26, 28].

It has been shown that prolonged administration of prophylactic doses of ML heartworm-preventive drugs, particularly ivermectin or moxidectin, kills older larvae, immatures (juveniles), young adults, and mature adults. In addition, a high percentage of dogs become amicrofilaremic within a few weeks to several months after dosing is started. The rate of kill with such treatment with ivermectin is dependent on the age of the heartworms when monthly treatment is initiated, with 3-month-old larvae requiring up to 1 year and mature adults requiring 2.5 years to provide efficacy of at least 95%. These data have been summarized [29]. More recently, it has been shown that the administration of doxycycline plus prophylactic doses of ivermectin has a synergistic effect on mf and adult heartworms [21]. Circulating mf were eliminated more quickly when both drugs were administered (by 12 weeks) than when the drugs were administered alone (few to many months) in most dogs, and adult worm efficacy was much higher at 36 weeks when both drugs were administered together (78.3%) than when doxycycline (8.7%) or ivermectin (20.3%) was given alone [22].

The American Heartworm Society recommends the initiation of daily doses of doxycycline for 1 month with a 1-month rest period and concurrently an ML preventive 2 months before the three-injection protocol for melarsomine is started, with administration of both drugs starting on the same day [30, 31]. The ML is expected to prevent further infection and gradually reduce microfilaremia levels. However, circulating mf can be detected in some dogs for several months after starting the administration of doxycycline or ivermectin alone or doxycycline plus an ML [22, 23, 25]. Monthly prophylactic doses of ivermectin along with a month of daily doses of doxycycline administered orally at 10 mg/kg twice daily (bid) can significantly reduce *Wolbachia* levels, reduce pulmonary infiltrates, reduce worm biomass, decrease post-treatment complications and morbidity [30, 31], and impede the spread of both ML-resistant and ML-susceptible heartworms [22, 23]. The additional 1-month (no-treatment) rest period is hypothesized to allow for the clearance of metabolites such as *Wolbachia* surface protein (WSP) and further reduction in the biomass before administration of melarsomine is started [30–32].

McCall et al. [22] demonstrated that treatment of *D. immitis* microfilaremic dogs with both doxycycline, administered orally at 10 mg/kg daily in weeks 1–6, 10–11, 16–17, 22–25, and 28–30, and ivermectin, administered orally at 6 mcg/kg once weekly until necropsy at 36 weeks, cleared mf by 12 weeks, with no rebound in mf, and killed 78.3% of the adult heartworms. The present study was designed to monitor mf counts over a longer duration of 647 days (21.3 months) for any rebound that might occur in mf counts and assess adulticidal efficacy following administration of doxycycline and ivermectin at the same dosages as in the earlier study but in two shorter treatment regimens.

## Methods

### General study design

Twelve adult beagle dogs were infected with 10 pairs of adult male and female *D. immitis* (Berkeley isolate), one male and one female per pair, by intravenous (IV) transplantation via a jugular vein [33]. After mf counts were greater than 1000 mf/ml, the dogs were randomly allocated to three groups of four dogs each based on mf count. Day 0 was the first day of dosing. Starting on day 0, Group 1 (short-treatment regimen) received doxycycline orally at approximately 10 mg/kg once daily (sid) for 30 days plus a heartworm-preventive dose of ivermectin (minimum, 6 mcg/kg) on days 0 and 30; Group 2 (long-treatment regimen) dogs received doxycycline orally at approximately 10 mg/kg sid until each dog was mf-negative (range, 72–98 doses) and a preventive dose of ivermectin every other week for a total of 6–7 doses. Group 3 served as the untreated control. Mf counts (modified Knott test) were performed prior to the first day of treatment, at 1–2-week intervals during the first 4 months, and then monthly thereafter until necropsy. Heartworm antigen (Ag) tests (DiroCHEK™ Heartworm Antigen Test Kit, Zoetis, Inc., Kalamazoo, MI, USA) were performed on all dogs prior to treatment, again at days 70 and 154, and then on days 644 and 647. One untreated control dog was euthanized (with no worm count) on day 112 due to severe heartworm disease, and the remaining 11 dogs were necropsied for recovery and enumeration of adult heartworms on day 647.

### Mosquito strain and heartworm isolate

In 1972, the black-eyed Liverpool strain of *Aedes aegypti* used in this study was obtained by the University of Georgia (UGA) from Professor W.W. Macdonald in the Department of Parasitology and Entomology at the Liverpool School of Tropical Medicine, who had obtained it from West Africa in 1962 [34]. TRS Labs, Inc. (TRS) obtained the strain from the UGA in 1980. During the 50 years that the mosquitoes were maintained at UGA and then TRS, it is estimated that the strain was maintained through a total of 2009 generations. For further details about this strain, see McCall et al. [35] in this issue.

The Berkeley isolate of *D. immitis* was used in this study. The isolate was obtained by TRS from Berkeley County, South Carolina (USA) in April 2014 and was validated by testing positive for heartworm mf and antigen and by worm recovery at necropsy in December 2014. It is known to be susceptible to ML heartworm preventives [36].

### Animals and animal management

Twelve purpose-bred male (six) and female (six) beagles from a commercial supplier were used in this study. They ranged in age from 1.1 to 1.3 years on the day of infection and ranged from 6.5 to 11.0 kg on day 0. They were born and raised indoors in mosquito-proof facilities and were not treated with any heartworm preventive-drugs prior to the start of this study. They had negative test results for mf (modified Knott test) and adult heartworm Ag (DiroCHEK™ Heartworm Antigen Test Kit, Zoetis, Inc., Kalamazoo, MI, USA) on day 0 (first day of treatment) and were randomly allocated by a table of random numbers to three groups of four dogs each based on similar heartworm mf counts. The dogs were housed individually in 4 ft. by 5 ft. kennels during dosing. Thereafter, they were pair-housed by gender within groups, i.e., with access to their mate’s kennel. This study was approved by the TRS Labs’ Institutional Animal Care and Use Committee [AUP 20-06(5)] prior to the initiation of the study, and the dogs received humane care, with at least a once-daily health observation, throughout the study.

### Study drugs

Doxycycline hyclate was administered as one 100-mg tablet or two 50-mg capsules (Harris Pharmaceuticals) orally sid to achieve a dosage of approximately 10 mg/kg/day. The daily dose range for Group 1 treated dogs was 9.1–10.9 mg/kg and the daily dose range for Group 2 treated dogs was 9.6–15.4 mg/kg.

Ivermectin was administered orally as 68-mcg chewables (Iverhart^®^, Virbac, Fort Worth, TX) to achieve a minimum dose of 6.0 mcg/kg of ivermectin. Each treated dog received one chewable per dose. The daily dose range for Group 1 treated dogs was 6.2–7.4 mcg/kg and the dose range for Group 2 treated dogs was 6.5–10.5 mcg/kg).

### Statistical analysis

Mean mf counts between experimental groups over the course of the study were compared using a two-tailed Wilcoxon matched-pairs signed-rank test with *α* = 0.05. A log-rank test was used to compare the time to mf clearance between short- and long-treatment regimen groups.

The numbers of adult male and female worms recovered at necropsy in the short-treatment and long-treatment regimen groups were compared to those of the control group using *t*-tests corrected with the Holm-Šídák method. All analyses were performed in GraphPad Prism 8.0 (GraphPad Software, La Jolla, CA, USA).

## Results

### Effects of treatment on mf counts

**Fig. 1:**
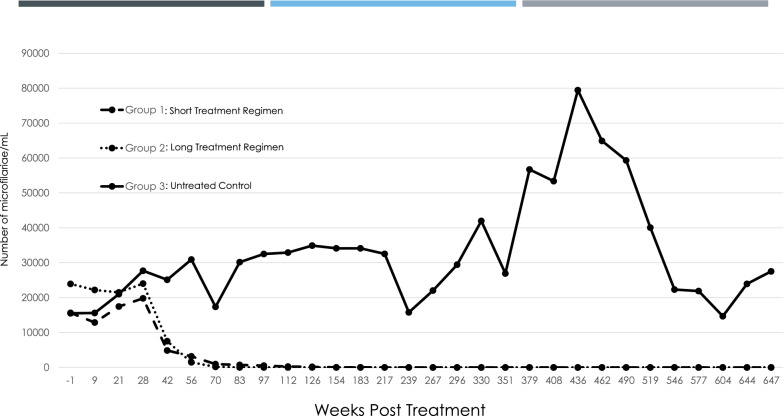
Mean microfilarial counts for dogs. All treatments started on day 0. Group 1 dogs (short-treatment regimen) were administered doxycycline orally at 10 mg/kg sid for 30 days plus ivermectin orally (minimum dose, 6 mcg/kg) on days 0 and 30. Group 2 dogs (long-treatment regimen) were administered doxycycline orally at 10 mg/kg sid until individual dogs became amicrofilaremic (days 72–98) and ivermectin every 2 weeks until individual dogs became amicrofilaremic (6–7 doses). Group 3 dogs were the untreated control. Group 1 and 2 dogs became microfilaria-negative on days 239 and 97, respectively, and remained negative through day 647. Significant differences in mf counts were observed between both treated Group 1 and treated Group 2 and the control (Group 3) (Wilcoxon matched-pairs signed-rank test, *P* < 0.0001 for each). No significant difference was observed between treated Groups 1 and 2 (Wilcoxon matched-pairs signed-rank test, *P* = 0.54). There was no significant difference in time to mf clearance between treated Groups 1 and 2, with a median time to clearance of 83.5 and 90 days, respectively (*χ*^2^ = 0.066, degrees of freedom [*df*] = 1, *P* = 0.80)

One untreated control dog (Group 3) was removed from the study on day 112, so mean counts from day 126 until the end of the study were based on values for the three remaining dogs. One day prior to the start of treatment on day 0, mean mf counts were 15,613, 23,950, and 15,513 mf/ml for Groups 1 (short-treatment regimen), 2 (long-treatment regimen), and 3 (untreated control), respectively (Fig. 1). Mean mf counts in the treated groups either increased slightly (Group 1) or remained the same (Group 2) until day 28. During the next 2 weeks, the mean mf counts in the two treated groups (Groups 1 and 2) declined rapidly and were 4863 and 7525 mf/ml on day 42, respectively, compared with a control count of 25,113 mf/ml at this time. In Group 1 (short-treatment regimen), one dog became mf-negative on day 42, a second dog became mf-negative on day 56, a third dog became mf-negative on day 97, and the fourth dog became mf-negative on day 239. Thus, all dogs in Group 1 were amicrofilaremic by day 239 and remained amicrofilaremic throughout the remainder of the study. In Group 2 (long-treatment regimen), one dog was mf-negative on day 70, a second dog became mf-negative on day 83, and the third and fourth dogs became mf-negative on day 97. Thus, all dogs in this group became amicrofilaremic by day 97 and remained amicrofilaremic throughout the remainder of the study. In contrast, all untreated control dogs (Group 3) had circulating mf throughout the study. Mean mf counts in this group gradually increased, with a mean mf peak count of 79,433 mf/ml on day 436. Thereafter, the mean mf count gradually decreased to 14,683 mf/ml on day 604 and then rose to 27,517 mf/ml just prior to necropsy on day 647 (21.3 months).

Significant differences in mf counts were observed between both the short-treatment (mean = 2634 mf/ml) and long-treatment (mean = 3477 mf/ml) regimen groups and the untreated control group (mean = 32,782 mf/ml; Wilcoxon matched-pairs signed-rank test, *P* < 0.0001 for each). No significant difference was observed between the short- and long-treatment regimen groups (Wilcoxon matched-pairs signed-rank test, *P* = 0.54).

A log-rank test found no significant differences in time to mf clearance between the short- and long-treatment regimen groups, with a median time to clearance of 83.5 and 90 days, respectively (*χ*^2^ = 0.066, *df* = 1?, *P* = 0.80).

### Adult heartworm antigen levels

As indicated in Table 1, heartworm Ag tests were conducted on days −1, 70, 154, 642, and 647. All of the dogs in Group 1 (short-treatment regimen) and Group 3 (untreated control) were antigen-positive on all of the tests. For Group 2 (long-treatment regimen), all of the dogs were antigen-positive on days −1, 70, and 154, but all were negative on days 642 and 647.

**Table 1:** Live adult heartworm recovery with efficacy and antigen test results in dogs treated with doxycycline and ivermectin, and untreated dogs

	No. of live adult *D. immitis*					Ag test results (±) on study day					
Group	An no.^a^	M	F	Total	Mean no. (total)	% Effic.	−1	70	154	624	647
1	9408	4	1	5			+	+	+	+	+
	9425	5	2	7	6.8	57.5%	+	+	+	+	+
	9417^b^	6	2	8			+	+	+	+	+
	9428^c^	5	2	7			+	+	+	+	+
2	9316	6	0	6			+	+	+	−	−^d^
	9411	1	0	1	3.3	79.4%	+	+	+	−	−^d^
	9423	1	0	1			+	+	+	−	−^d^
	9413	5	0	5			+	+	+	−	−^d^
3	9406	9	5	14			+	+	+	+	+
	9413	9	8	17	16.0	N/Ad	+	+	+	+	+
	9429	8	9	17			+	+	+	+	+
	9409	–	–	Euth. day 112			+	+	N/A^e^	N/A^e^	N/A^e^

All treatments started on day 0. Group 1 dogs (short-treatment regimen) were administered doxycycline orally at 10 mg/kg sid for 30 days plus ivermectin orally (minimum dose, 6 mcg/kg) on days 0 and 30. Group 2 dogs (long-treatment regimen) were administered doxycycline orally at 10 mg/kg sid until individual dogs became amicrofilaremic (72–98 days) and ivermectin every 2 weeks until individual dogs became amicrofilaremic (6–7 doses). Group 3 dogs were the untreated control. Group 1 and 2 dogs became mf-negative on days 239 and 97, respectively, and remained negative through day 647. The numbers of live male and female worms from treated Group 1 were significantly fewer than from the untreated control (Group 3) [*t*-test, *t*_(5)_ = 6.574, *P* < 0.001 and *t*_(5)_ = 5.327, *P* < 0.004, respectively]. Similarly, significantly fewer male and female worms were recovered from treated Group 2 compared to the control (Group 3) [*t*-test, *t*_(5)_ = 3.427, *P* < 0.019 and *t*_(5)_ = 7.293, *P* < 0.0009, respectively]

^a^Infected IV with 10 male and 10 female adult heartworms per dog

^b^One dead female worm

^c^Two dead female worms

^d^Same results for unheated and preheated samples

^e^*N/A* not applicable

### Recovery of heartworms at necropsy

As shown in Table 1, all dogs were infected by IV transplantation of 10 pairs of adult male and female heartworms, with each pair consisting of one male and one female worm (total, 20 heartworms/dog). At necropsy, one or two dead female worms were recovered from two dogs in Group 1. No dead worms were recovered from dogs in Groups 2 and 3. In regard to live heartworms, all dogs in Group 1 (short-treatment regimen) had live male and female heartworms, with a mean of 6.8 worms per dog and a range of 5–8. All dogs had one or two live female heartworms plus at least four live male worms (range, 4–6). Adult worm efficacy for this group was 57.5%. For Group 2 (long-treatment regimen), none of the four dogs had any live adult female heartworms, while all of the dogs had live adult male heartworms, with a mean of 3.3 worms per dog (range, 1–6 worms/dog). Adult worm efficacy for this group was 79.4%. In comparison, all of the three untreated control dogs (Group 3) had live adult heartworms, with a mean of 16.0 worms per dog (range, 14–17). Each dog in this group had at least eight male and five female heartworms.

The numbers of adult male and female worms recovered from animals in the short-treatment regimen group (Group 1) were significantly fewer than those from the untreated control group (Group 3) [*t*-test, *t*_(5)_ = 6.574, *P* < 0.001 and *t*_(5)_ = 5.327, *P* < 0.004, respectively]. Similarly, significantly fewer male and female worms were recovered from the long-treatment regimen group (Group 2) compared to the control group (Group 3) [*t*-test, *t*_(5)_ = 3.427, *P* < 0.019 and *t*_(5)_ = 7.293, *P* < 0.0009, respectively].

## Discussion

For heartworm adulticidal therapy, the American Heartworm Society currently recommends the administration of daily, oral doses of doxycycline administered at 10 mg/kg bid for 28 days and an ML heartworm-preventive drug, with dosing of both drugs starting on the same day 2 months before the first injection of the three-injection protocol for melarsomine is given [31]. While the doxycycline plus ML treatment will eventually clear mf from the blood, some mf have been shown to persist for up to 3 weeks following administration of high-dose topical moxidectin [1.0 ml or 2.5 ml of imidacloprid (IMD) + moxidectin by labeled microfilaricidal dosage and administration for 10 months] plus oral doxycycline (10.0–14.1 mg/kg, minimum, 10 mg/kg bid) for 30 days) [25] and up to 5.5 months following initiation of daily doses of doxycycline (10 or 5 mg/kg bid for 28 days) and six monthly prophylactic doses of ivermectin plus pyrantel pamoate (minimum, 6 mcg/kg ivermectin by label dosage administration) [26]. In both of these studies, once all of the dogs in the treated groups became amicrofilaremic, there was no rebound in circulating mf thereafter. However, examination for mf in both of these studies was discontinued relatively early (5.5–8.2 months) after dosing was started. It seems possible that a rebound might have been detected if the studies had been continued for a longer period. In the current study, which used half the dosage of doxycycline and shorter ML treatment periods, circulating mf continued to persist for 239 days in one of the four dogs after the short-treatment regimen of doxycycline (30 days), and two monthly prophylactic doses of ivermectin were initiated. Moreover, mf continued to circulate for up to 97 days in one of the four dogs on the long-treatment regimen of doxycycline and 6–7 biweekly prophylactic doses of ivermectin. However, once the treated dogs became amicrofilaremic, there was no rebound in circulating mf for the remainder of the 647-day (20.3 months) study. These observations indicate that even the low dosages and short-treatment regimen of doxycycline and ivermectin used in the short-treatment regimen group, which received less doxycycline (i.e., one-half the daily dosage and a similar treatment length) and the same dosage in a shorter ML treatment regimen than the Savadelis et al. study [26], were sufficient to prevent a rebound in mf counts for over 20 months. This indicates that the current recommendations to repeat doxycycline dosing after 1 year in slow-kill protocols [37] will not be needed to prevent a rebound in circulating mf but may be necessary in antigen-positive dogs to kill persisting adult heartworms. The earlier clearance of mf in the long-treatment regimen group in the current study can be attributed to the longer treatment with doxycycline and ivermectin. The earlier clearance of circulating mf in the Savadelis et al. [25] study than in the current study can be attributed to the use of high-dose doxycycline plus high-dose moxidectin, which is an approved heartworm microfilaricide [38].

In regard to efficacy against adult heartworms, mean recoveries for the short-treatment regimen (Group 1), long-treatment regimen (Group 2) and untreated controls (Group 3) were 6.8, 3.3, and 16.0, respectively (Table 1). Adult worm recoveries were significantly reduced in both the short- and long-treatment regimen groups when compared to the untreated controls. Moreover, worm recoveries in the two treated groups were substantially, but not statistically, different from each other. In addition, there was a gender effect in the two treated groups. No live female worms were recovered from dogs in the long-treatment regimen group, whereas all dogs in the short-treatment regimen group had one or two live female worms. Also, about twice as many male worms were recovered from the short-treatment regimen group as from the long-treatment regimen group. Considering the larger size, more worm tissue invaded and destroyed by the *Wolbachia*, and presumably more dead *Wolbachia* in female worms than in male worms [9, 39], it seems reasonable to assume that the longer treatment regimen resulted in a higher death rate of *Wolbachia*, which is generally considered necessary for long-term survival of the worms. This probably led to a higher death rate in the female worms. Considering that there are more *Wolbachia* in a female worm than a male worm and observing a higher death rate in the female worms strongly suggests that female worms are more dependent on *Wolbachia* than male worms.

It is noteworthy that no gender effect was noted in the Savadelis et al. [25] study. Moreover, adulticidal efficacy was higher in the Savadelis et al. [25] study than in the current study. It seems plausible that the difference in the two studies might be due to differences in the ML and dosage level/regimen length used, the dosage of doxycycline used, and/or the duration of the study. In that study, a total of only one male worm was recovered from the treated group, while a total of four females were recovered and the overall efficacy of treatment was 95.9%. The Savadelis et al. [25] study used a higher doxycycline dosage of 10 mg/kg bid for 30 days and more (10 monthly) treatments of the ML (high-dose, microfilaricidal moxidectin), while a lower dosage of 10 mg/kg sid for 30 days and two monthly doses of low-dose (prophylactic) ivermectin (short-treatment regimen) or a lower dosage of 10 mg/kg sid for 72–98 days and 6–7 biweekly doses of low-dose (prophylactic) ivermectin (long-treatment regimen) were used in the current study. Adulticidal efficacy in the current study was 57.5% for the low-dose, short-treatment regimen group compared to 79.4% for the low-dose, long-treatment regimen group. The higher adulticidal efficacy obtained with the longer treatment regimen for both drugs in the current study can be attributed to the more drug given for a longer period of time. The higher adulticidal efficacy seen in the Savadelis et al. [25] study was probably due to the use of a higher dosage of doxycycline and high-dose moxidectin.

## Conclusions

Even when low dosages and short-treatment regimens of both doxycycline (10 mg/kg sid for 30 days) and ivermectin (minimum dose of 6 mcg/kg on days 0 and 30) were administered to heartworm-microfilaremic dogs (Group 1), once the dogs became mf-negative, there was no rebound in the count for at least 647 days (21.3 months). And with the long-treatment regimen of both doxycycline (10 mg/kg sid daily) and ivermectin (minimum dosage of 6 mcg/kg every other week until each dog became amicrofilaremic), there was complete elimination of adult female heartworms and a substantial reduction in male heartworms at necropsy. The earlier clearance of mf from the blood and the higher adulticidal efficacy obtained with the longer treatment regimen for both drugs in the current study can be attributed to more of both drugs being given and the long holding period after treatment.

The data in this study lend further support to the use of the American Heartworm Society Canine Guidelines for adulticidal therapy recommendation of initiating doxycycline plus an ML treatment at the same time as the heartworm-positive diagnosis.

## Data Availability

Not applicable.

## Abbreviations

ML: Macrocyclic lactone
mf: Microfilaria, microfilariae, microfilarial
Ag: Antigen
L_3_: Infective third-stage larvae
L_4_: Fourth-stage larvae
sid: Once daily
bid: Twice daily
UGA: University of Georgia
TRS: TRS Labs, Inc.
IMD: Imidacloprid

## References

[1] Sironi M, Bandi C, Sacchi L, DiSacco B, Damini G, Genchi C (1995). A close relative of the arthropod endosymbiont *Wolbachia* in a filarial worm. Mol Biochem Parasitol.

[2] Genchi C, Sacchi L, Bandi C, Venco L (1998). Preliminary results on the effect of tetracycline on the embryogenesis and symbiotic bacteria (*Wolbachia*) of *Dirofilaria immitis*: an update and discussion. Parasitologia.

[3] Bandi C, McCall JW, Genchi C, Corona S, Venco L, Sacchi L (1999). Effects of tetracycline on the filarial worms *Brugia pahangi* and *Dirofilaria immitis* and their bacterial endosymbionts *Wolbachia*. Int J Parasitol.

[4] Bandi C, Trees AJ, Brattig NW (2001). *Wolbachia* in filarial nematodes: evolutionary aspects and implications for the pathogenesis and treatment of filarial diseases. Vet Parasitol.

[5] Casiraghi M, Anderson TJC, Bandi C, Bazzocchi C, Genchi C (2001). A phylogenetic analysis of filarial nematodes: comparison with the phylogeny of *Wolbachia* endosymbionts. Parasitology.

[6] Casiraghi M, Bain O, Guerrero R, Martin C, Pocacqua V, Gardner SI (2004). Mapping the presence of *Wolbachia pipientis* on the phylogeny of filarial nematodes evidence for symbiont loss during evolution. Int J Parasitol.

[7] Taylor MJ, Bandi C, Hoerauf A (2005). *Wolbachia* bacterial endosymbionts of filarial nematodes. Adv Parasitol.

[8] McCall JW, Genchi C, Kramer L, Guerrero J (2008). Heartworm disease in animals and humans. Adv Parasitol.

[9] Fenn K, Baxter M (2004). Quantification of *Wolbachia* bacteria in *Brugia malayi* through the nematode lifecycle. Mol Biochem Parasitol.

[10] Bosshardt SC, McCall JW, Coleman SU, Jones KL, Petit TA, Klei TR (1993). Prophylactic activity of tetracycline against *Brugia pahangi* infection in jirds (*Meriones unguiculatus*). J Parasitol.

[11] Hoerauf A, Nissen-Pahle K, Schmetz C, Henkle-Duhrsen K, Blaxter ML, Buttner DW (1999). Tetracycline therapy targets intracellular bacteria in the filarial nematode *Litomosoides sigmodontis* and results in filarial infertility. J Clin Invest.

[12] Hoerauf A, Volkmann L, Hamelmann C, Adjei O, Autenrieth IB, Fleischer B (2000). Endosymbiotic bacteria in worms as targets for a novel chemotherapy in filariasis. Lancet.

[13] Hoerauf A, Mand S, Volkmann L, Buttner M, Marfo-Debrekyer Y, Taylor M (2003). Doxycycline in the treatment of human onchocerciasis: kinetics of *Wolbachia* endo-bacteria reduction and inhibition of embryogenesis in female *Onchocerca* worms. Microbes Infec.

[14] Hoerauf A, Mand S, Fischer K, Kruppa T, Marfo-Debrekyei Y, Debrah AY (2003). Doxycycline as a novel strategy against bancroftian filariasis—depletion of *Wolbachia* endosymbionts from *Wucherreria bancrofti* and stop of microfilaria production. Med Microbiol Immunol.

[15] Townson S, Sutton D, Stemenski J, Hollick L, Scanlon T, Tagoboto SK (2000). Antibiotics and *Wolbachia* in filarial nematodes: antifilarial activity of rifampicin, oxytetracycline and chloramphenicol against *Onchocerca gutturosa, Onchocerca lienalis* and *Brugia pahangi*. Ann Trop Med Parasitol.

[16] McCall JW, Jun JJ, Bandi C (1999). *Wolbachia* and the antifilarial properties of tetracycline: an untold story. Ital J Zool.

[17] Langworthy N, Renz A, Meckenstedr U, Henkle-Duhrsen K, Bronvoort M, Tanya V (2000). Microfilaricidal activity of tetracycline against the filarial nematode *Onchocerca ochengi*: elimination of *Wolbachia* precedes worm death and suggests a dependent relationship. Proced Roy Soc London Series B Biolog Sci.

[18] Taylor MJ, Makunde WH, McGarry HF, Turner JD, Mand S, Hoerauf A (2005). Macrofilaricidal activity after doxycycline treatment of *Wuchereria bancrofti*: a double-blind, randomized placebo-controlled trial. Lancet.

[19] Bazzocchi C, Mortarino M, Grandi G, Kramer LH, Genchi C, Bandi C (2008). Combined ivermectin and doxycycline treatment has microfilaricidal and adulticidal activity against *D. immitis* in experimentally infected dogs. Int J Parasitol.

[20] Chandrashekar R, Beall MJ, Saucier J, O’Connor T, McCall JW, McCall SD (2014). Experimental *Dirofilaria immitis* infection in dogs: effects of doxycycline and advantage multi^®^ administration on immature adult parasites. Vet Parasitol.

[21] Grandi G, Quintavalla C, Mavropoulou A, Genchi M, Gnudi G, Bertoni G (2010). A combination of doxycycline and ivermectin is adulticidal in dogs with naturally acquired heartworm disease (*Dirofilaria immitis*). Vet Parasitol.

[22] McCall JW, Genchi C, Kramer L, Guerrero J, Dzimianski MT, Supakorndej P (2008). Heartworm and *Wolbachia*: therapeutic implications. Vet Parasitol.

[23] McCall JW, Kramer L, Genchi C, Guerrero J, Dzimianski MT, Mansour A (2014). Effects of doxycycline on heartworm embryogenesis, transmission, circulating microfilaria, and adult worms in microfilaremic dogs. Vet Parasitol.

[24] Kramer L, Grandi G, Leoni M, Passeri B, McCall J, Genchi C (2008). *Wolbachia* and its influence on the pathology and immunology of *Dirofilaria immitis* infection. Vet Parasitol.

[25] Savadelis MD, Ohmes CM, Hostetler JA, Settje TL, Zolynas R, Dzimianski MT (2017). Assessment of parasitological findings in heartworm-infected beagles treated with advantage multi^®^ for dogs (10% imidacloprid + 2.5% moxidectin) and doxycycline. Parasit Vectors.

[26] Savadelis MD, Day KM, Bradner JL, Wolstenholme AJ, Dzimianski MT, Moorhead AR (2018). Efficacy and side effects of doxycycline versus minocycline in the three-dose melarsomine canine adulticidal heartworm treatment protocol. Parasit Vectors.

[27] McCall JW, Kramer L, Genchi C, Guerrero J, Dzimianski MT, Supakorndej P (2011). Effects of doxycycline on early infections of *Dirofilaria immitis* in dogs. Vet Parasitol.

[28] Grandi G, Kramer L, Bazzocchi C, Mortarino M, Venco L, Genchi C (2005). *Dirofilaria immitis* and the endosymbiont *Wolbachia*: Inflammation and immune response in heartworm disease. Jpn J Vet Parasitol.

[29] McCall JW (2005). The safety-net story about macrocyclic lactone heartworm preventives: a review, an update and recommendations. Vet Parasitol.

[30] Nelson CT, McCall JW, Jones S, Moorhead A (2018). Current canine guidelines for the prevention, diagnosis, and management of heartworm infection in dogs.

[31] Nelson CT, Myrick ES, Nelson TA (2017). Clinical benefits of incorporating doxycycline into a canine heartworm treatment protocol. Parasit Vectors.

[32] McCall JW. History of canine heartworm treatment and rationale for current AHS recommendations. In: Payne, P., McCall, J.W. (eds). Bulletin, American Heartworm Society, Wilmington, Delaware. 2018;45(1):9–15.

[33] Rawlings CA, McCall JW (1982). Surgical implantation of adult *Dirofilaria immitis* to study heartworm infection and disease in dogs. Am J Vet Res.

[34] Macdonald MM (1962). The selection of a strain of *Aedes aegypti* susceptible to infection with semi-periodic *Brugia malayi*. Ann Trop Med Parasitol.

[35] McCall JW, DiCosty U, Mansour A, Fricks C, McCall S, Dzimianski MT, et al. Inability of *Dirofilaria immitis* infective larvae from mosquitoes fed on blood from microfilaremic dogs during low-dose and short-treatment regimen doxycycline and ivermectin treatment to complete normal development in heartworm naïve dogs. Parasit Vectors. 2023; In press. 10.1186/s13071-023-05704-5 PMC10262448 37312202

[36] Baker C, Tielemans E, Pollmeier MG, McCall JW, McCall SD, Chester TS (2014). Efficacy of a single dose of a novel topical combination product containing eprinomectin to prevent heartworm infection in cats. Vet Parasitol.

[37] Jacobson LS, DiGangi BA (2021). An accessible alternative to melarsomine: “Moxi-Doxy” for treatment of adult heartworm infection in dogs. Front Vet Sci.

[38] McCall JW, Arther R, Davis W, Setje T (2014). Experimental *Dirofilaria immitis* infection in dogs. Vet Parasitol.

[39] McGarry HG, Egerton G, Taylor MJ (2004). Population dynamics of *Wolbachia* bacterial endosymbionts in *Brugia malayi*. Mol Biochem Parasitol.

